# Female developmental environment delays development of male honeybee (*Apis mellifera*)

**DOI:** 10.1186/s12864-021-08014-1

**Published:** 2021-09-27

**Authors:** Yi Bo Liu, Yao Yi, Amal Abdelmawla, Yun Lin Zheng, Zhi Jiang Zeng, Xu Jiang He

**Affiliations:** 1 Honeybee Research Institute, Jiangxi Agricultural University, 330045 Nanchang, China; 2 Jiangxi Key Laboratory of Honeybee Biology and Bee Keeping, 330045 Nanchang, Jiangxi China; 3 Jiangxi University of Traditional Chinese Medicine, Jiangxi 330004 Nanchang, P. R. China

**Keywords:** Honeybee, Larval diets, Cell sizes, Male development, Sexual differentiation, Gene expression

## Abstract

**Background:**

Nutrition and cell size play an important role in the determination of caste differentiation in queen and worker of honeybees (*Apis mellifera*), whereas the haploid genome dominates the differentiation of drones. However, the effects of female developmental environment on the development of males remain unclear. In this study, young drone larvae were transferred into worker cells (WCs) or remained in drone cells (DCs) to rear drones. The drone larvae were also grafted into queen cells (QCs) for 48 h and then transplanted into drone cells until emerging. Morphological indexes and reproductive organs of these three types of newly emerged drones were measured. Newly emerged drones and third instar drone larvae from WCs, DCs and QCs were sequenced by RNA sequencing (RNA-Seq).

**Results:**

The amount of food remaining in cells of the QC and WC groups was significantly different to that in the DC group at the early larval stage. Morphological results showed that newly emerged DC drones had bigger body sizes and more well-developed reproductive tissues than WC and QC drones, whereas the reproductive tissues of QC drones were larger than those of WC drones. Additionally, whole body gene expression results showed a clear difference among three groups. At larval stage there were 889, 1761 and 1927 significantly differentially expressed genes (DEGs) in WC/DC, QC/DC and WC/QC comparisons, respectively. The number of DEGs decreased in adult drones of these three comparisons [678 (WC/DC), 338 (QC/DC) and 518 (WC/QC)]. A high number of DEGs were involved in sex differentiation, growth, olfaction, vision, mammalian target of rapamycin (mTOR), Wnt signaling pathways, and other processes.

**Conclusions:**

This study demonstrated that the developmental environment of honeybee females can delay male development, which may serve as a model for understanding the regulation of sex differentiation and male development in social insects by environmental factors.

**Supplementary Information:**

The online version contains supplementary material available at 10.1186/s12864-021-08014-1.

## Background

The environment plays instructive and important role in the regulation of animal development [1]. Diets, temperature, photoperiod, predators and other factors can modulate animal developmental trajectories [2–4]. This foundation is well known to entomologists. For instance, variations of nutrition and temperature lead to three different female castes in ants (*Myrmecina nipponica*): alate queens, wingless worker and wingless ergatoid queen [5]. Nutrition of honeybee (*Apis mellifera*) larvae mainly determines phenotypic plasticity of females, and royal jelly is necessary and sufficient for queen development [6]. However, if environmental factors affect sex differentiation and male development in honeybees remains unclear.

A honeybee (*Apis mellifera*) colony is composed of a queen, thousands of workers and several hundred drones, and is a classical model of understanding sociality and sex determination. The queen and workers are both females that develop from diploid eggs [7]. Several studies have reported that environmental factors including different diets and cell size between the queen and workers determine their dimorphism. During the whole larval period, queen larvae are fed with dramatically more food than worker and drone larvae [6]. The royal jelly for queen larvae contains 1:1 watery-clear and milky-white, whereas jellies for worker and drone larvae contain 3:1 or 4:1 of these two components [6]. Moreover, sugar, vitamin, amino acid, protein and nucleic acid levels are different in their larval diets [6, 8–11], which cause differences in physiology, behavior, gene expression and DNA methylation between the queen and workers [12–15]. The difference in their cell size also contributes to their caste differentiation [16].

Under normal conditions, honeybee drones arise from haploid eggs, which are laid in drone cells by queens. The complementary sex determiner (*csd*) gene acts as the primary signal of the sex-determining pathway in honeybees, initiating female development by *csd*-heterozygotes and male development in *csd*-homozygotes [17]. The *csd* gene controls the splicing of the feminizer gene (*fem*), and the *fem* gene regulates the expression of a sex-specific gene (doublesex, *dsx*) via alternative splicing [18].

However, few studies have investigated the effects of larval diet on drone development. The larval diet of drones is similar to a worker’s diet (worker jelly in the first three days and a yellowish pollen-containing food in the later three days) [6]. It is unclear whether there is a difference between the components of drone and worker larval diets. Feeding drone larvae with the food of worker larvae of corresponding age can produce normal adult drones [6, 19]. However, a previous study showed that workers that emerged from drone cells had larger acid glands and a higher number of ovarioles [20]. Drones developed from worker cells (WCs) have smaller body sizes [21], reflecting that the female developmental environment may affect drone development. In a hopelessly queen-less colony, ovaries of many workers enlarge and become functional. They start to lay thousands of unfertilized eggs in worker cells and many of them could successfully develop as drones [7, 22]. The population of the colony declines owning to death of old workers and the interrupted supply of new workers [22].

Drones play an important role in honeybee breeding, as their primary function is mating with virgin queens. Drones provide half of the DNA in breeding populations and are a source of genetic mutations in subsequent generations [7]. High-quality drones could provide more and high-quality sperms [23]. The existence of drones stimulates workers to forage more frequently and make the whole colony healthy [24]. Therefore, high-quality drones could broaden the genetic pool and increase the fitness of the whole colony.

Measurement of morphological indexes and reproductive tissues has been used for assessing drone quality, such as body size, the weight of mucus glands, seminal vesicles, the extent of hamuli, length of hind leg parts, total length of the hind leg and head width [25–27]. As only a few strongest drones (average 12) can successfully mate with a virgin queen during their mating flight, the flying, olfactory and visual abilities of drones are essential factors in assessing their quality [7]. According to Berg et al., the body size of drones is highly related to reproductive success [21]. Therefore, in this study, the morphological indexes and reproductive tissue sizes of newly emerged drones from worker cells (WCs), queen cells (QCs) and natural drone cells (DCs) were measured and compared. In addition, RNA-Seq provides a deep insight into the molecular mechanisms of honeybee queen-worker differentiation [28]. We therefore used RNA-Seq to identify the molecular mechanisms underpinning the effects of these different environments on drone development.

## Results

### Weight of larvae and larval food remaining in the cells of the three types

**Fig. 1:**
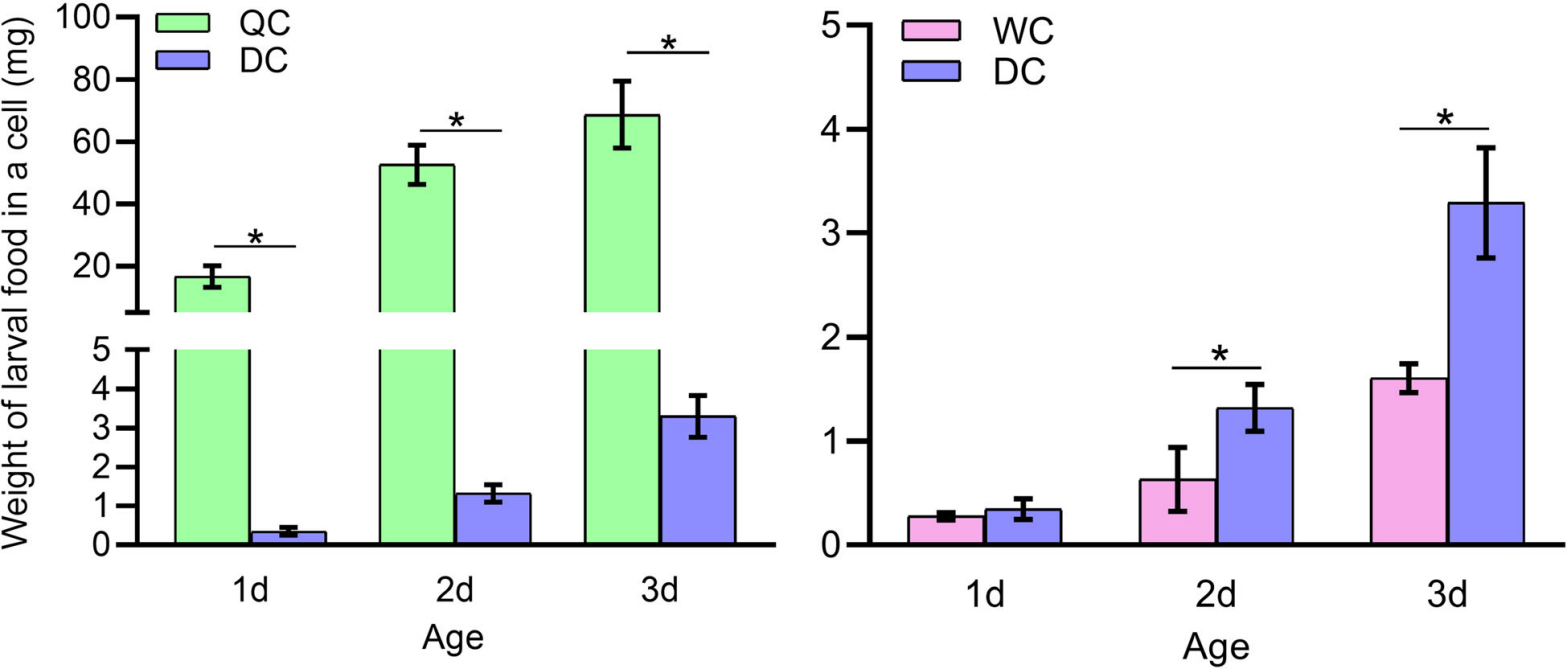
Weight of larval food remaining in queen, worker and drone cells. Mean ± SD were represented in all groups. Data were analyzed by one-way ANOVA test followed by Fisher’s PLSD test. Different characters on the top of bars represent significant difference (*p* < 0.025), same character indicates no difference (*p* > 0.025)

The weight of food remaining in queen cells of 1d, 2d and 3d QC drones was all significantly higher compared to that remaining in cells of DC drones, whereas the larval food remaining in worker cells of 2d and 3d WC drones was all significantly less compared to DC drones (Fig. 1).

### Morphology index and reproductive tissue analyses

**Fig. 2:**
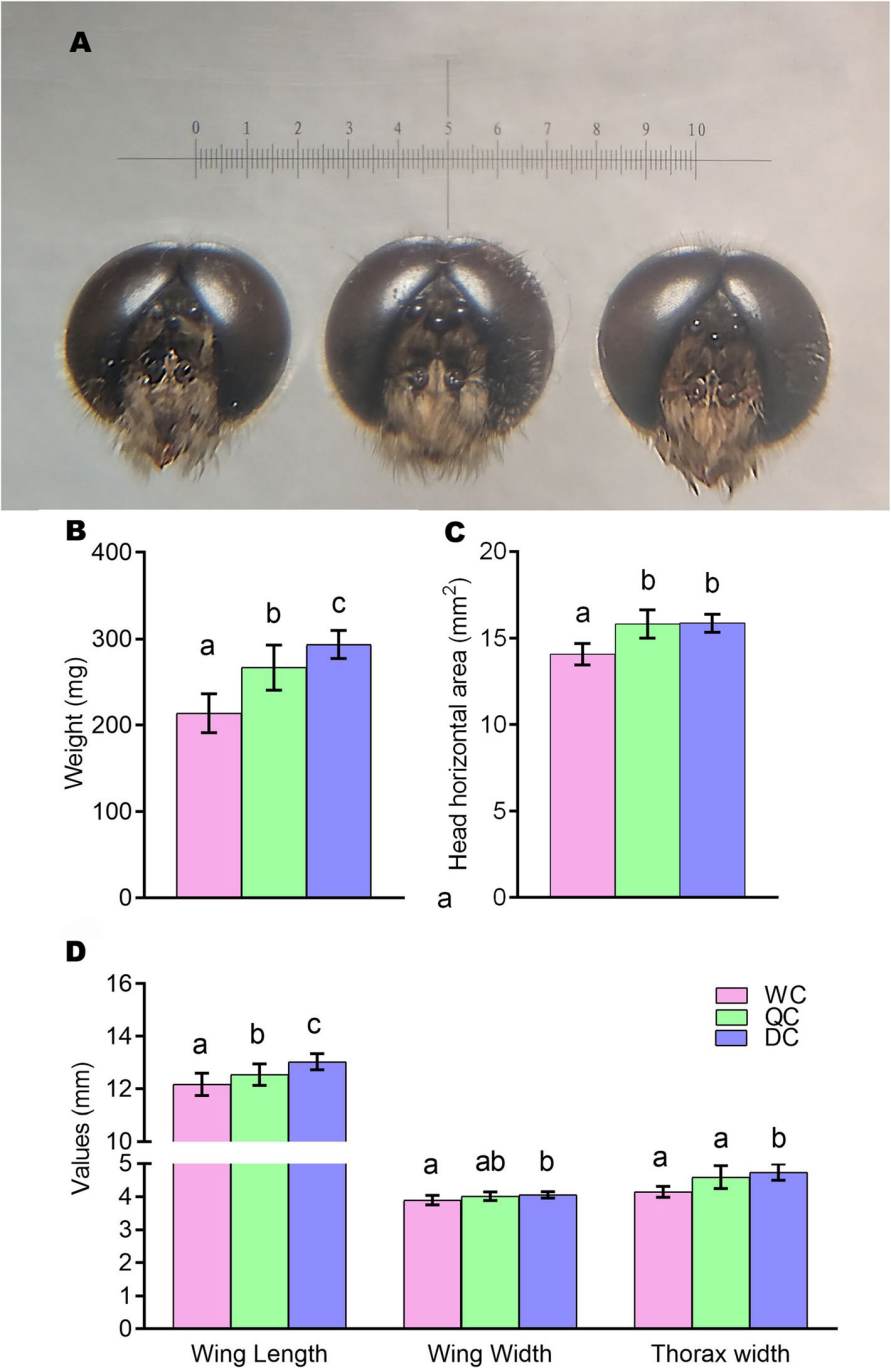
** A**: A view of heads from WCs, QCs and DCs under a microscope (from left to right: WCs, QCs and DCs; accuracy: 100 μm). B: Weight of newly emerged WCs, QCs and DCs. **C**: Head horizontal area of WCs, QCs and DCs. **D**: Thorax width, wing length and width of WCs, QCs and DCs. Mean ± SD were represented in all groups. Data were analyzed by Independent-Sample T test. The critical *p* values of were adjusted to 0.0167 according to the Bonferroni correction. Different characters on the top of bars represent significant difference (*p* < 0.0167), same character indicates no difference (*p* > 0.0167)

**Fig. 3:**
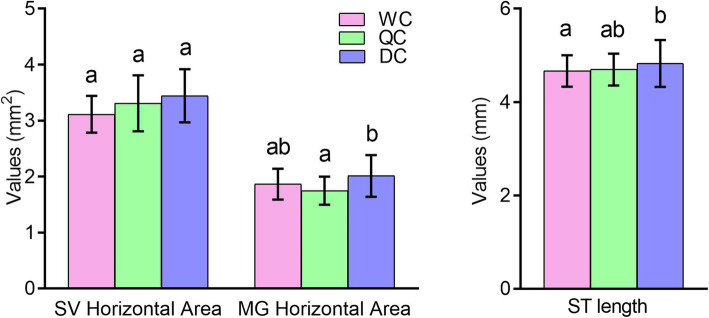
** A** Horizontal areas of seminal vesical (SV) and mucous glands (MG) of WCs, QCs and DCs. **B**: Length of seminiferous tubules (ST) of WCs, QCs and DCs. Mean ± SD were represented in all groups. Data were analyzed by Independent-Sample T test. The critical *p* values of were adjusted to 0.016 according to the Bonferroni correction. Different characters on the top of bars represent significant difference (*p* < 0.0167), same character indicates no difference (*p* > 0.0167)

Morphological results showed that newly emerged drone-cell drones (DC drones) had significantly larger body sizes and reproductive tissues than those of WC and QC drones (Figs. 2 and 3). In particular, the weight, wing length and width, thorax width and head horizontal area of DC drones were all significantly higher or larger than that of WC drones (Fig. 2B-D, *p* < 0.0167). The weight, wing length and thorax width of DC drones were significantly higher than the QC drones (Fig. 2B and D, *p* < 0.0167). The weight, wing length and head horizontal area of QC drones were significantly larger than those of WC drones (Fig. 2, p < 0.0167).

Considering reproductive organs, newly emerged DC drones had larger seminal vesicles than WC drones and QC drones, though there were no significant difference. And DC drones had significantly larger mucous glands than QC drones (Fig. 3 A, p < 0.0167). DC drones also had a longer length of seminiferous tubules than WC drones (Fig. 3B, *p* < 0.0167).

### Quality of RNA sequencing (RNA-Seq) data

Transcriptome analysis was done with 18 samples including three third instar larval groups and three adult groups (each group had 3 replicates). In total, 153.90 GB of clean data was obtained. The clean data for each sample was 6.17 GB, and the percentage of Q30 bases was 92.24 % or above (Table S1). After quality filtering and adapter trimming, the number of clear reads per sample ranged from 41 to 97 million, with an average of 57 million reads per sample. The proportions of mapped reads to clean reads ranged from 94.44 to 97.09 % (Table S2). These results revealed that the RNA-Seq quality of all samples was high, and their data were reliable. Pearson’s correlation coefficient of all biological replicates in each group was above 0.8, except for one sample from DC larvae (Fig. S1).

### Differentially expressed genes (DEGs)

At the adult stage, WC/DC comparison had the highest number of DEGs (678), followed by QC/WC (518) and QC/DC (338) comparisons (Table 1). However, at the 3rd instar stage, QC/WC comparison had the highest number of DEGs (1927), followed by QC/DC (1761) and WC/DC (889) comparisons (Table 1).

**Table 1:** Number of DEGs in three comparisons at 3rd instar and newly emerged stages

Sample comparisons	Upregulated	Downregulated	Total
WC larvae VS DC larvae	685	204	889
QC larvae VS DC larvae	950	811	1761
WC larvae VS QC larvae	783	1144	1927
WC adults VS DC adults	178	500	678
QC adults VS DC adults	79	259	338
WC adults VS QC adults	208	310	518

DEGs were identified when they had *p*-value < 0.05 and Fold Change ≥ 1. Up- and down-regulation of DEGs was determined by whether log fold change was above or below zero, respectively

**Fig. 4:**
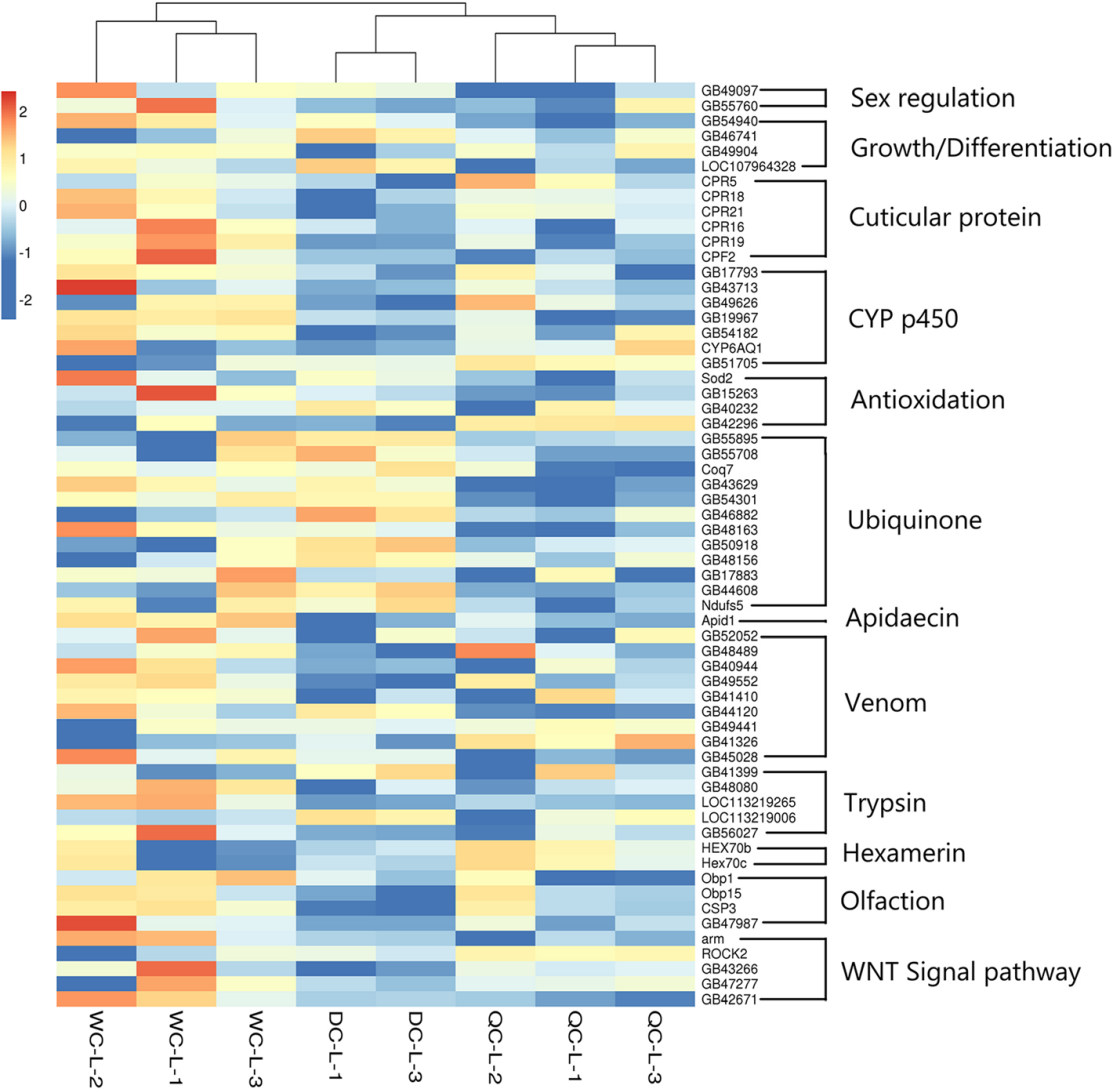
Expression of 61 selected DEGs among of third instar larvae of WCs, QCs and DCs. The log10 fold change value of each selected gene in each larval sample was used for analysis and presented with color scales. Data were analyzed by a Heatmap analysis in R package (4.0.2)

**Fig. 5:**
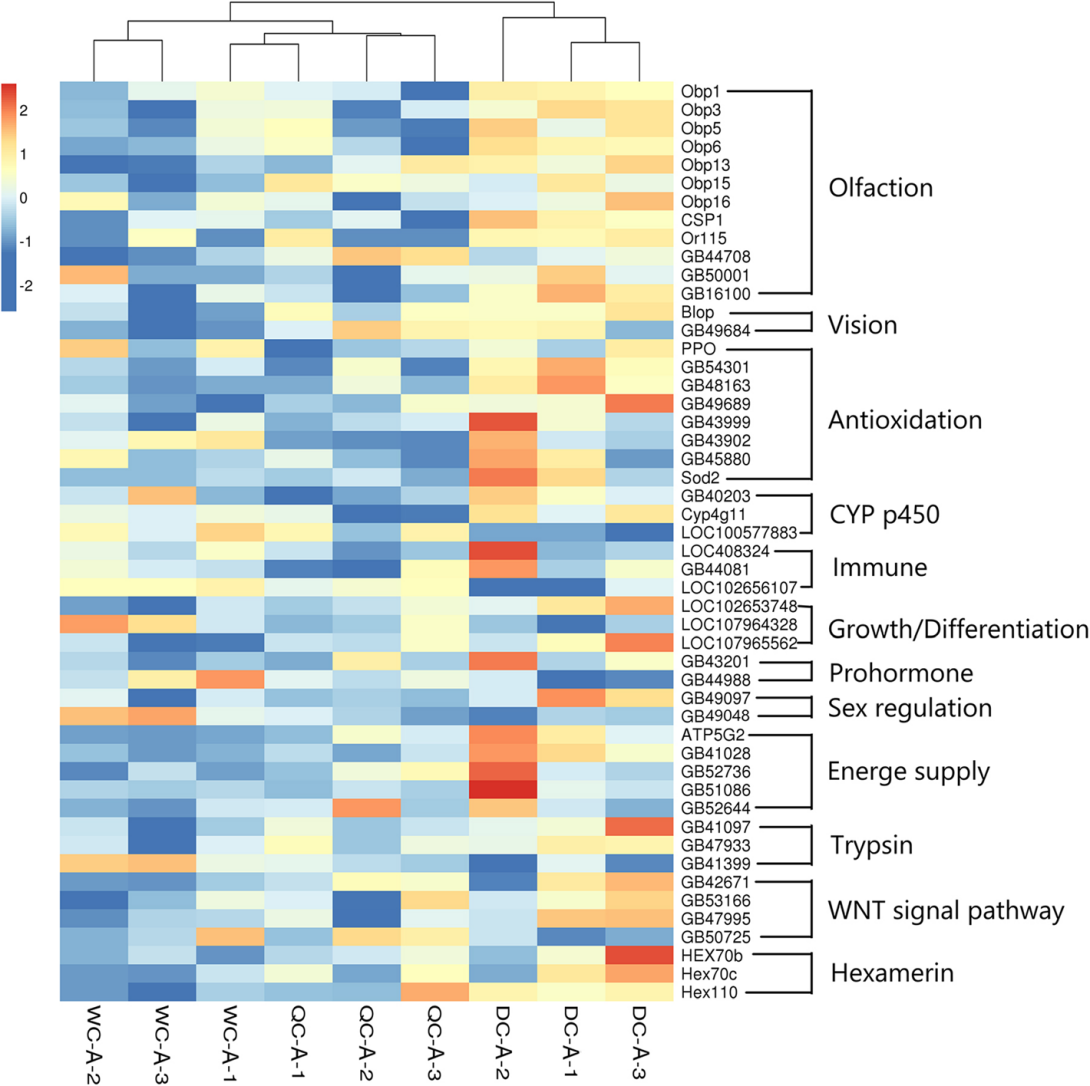
Expression of 50 selected DEGs among WC, QC and DC adult drones. The log10 fold change value of each selected gene in each adult drone sample was used for analysis and presented with color scales. Data were analyzed by a Heatmap analysis in R package (4.0.2)

A high number of DEGs were involved in honeybee drone growth and development, metabolism, immune and caste differentiation (Figs. 4 and 5; Table S3, 4, 5, 6). Therefore, we selected 61 DEGs, which are involved in caste differentiation and development regulation [30–36] among three larval groups. As shown in Fig. 4, DC larvae had a different expression pattern, compared with the QC and WC larvae. In contrast, WC drones had a different gene expression, compared with QC and DC ones (Fig. 5).

According to quantitative real-time reverse transcription PCR (qRT-PCR), the expression of most (8/10) of a set of randomly-selected genes was consistent with the RNA-Seq results in both 3rd instar and newly emerged drones (Fig. S2 and S3), reflecting that the RNA-Seq data are credible.

### Gene Ontology (GO) and Kyoto Encyclopedia of Genes and Genomes (KEGG) pathway enrichment analysis

The DEGs from DC/QC and DC/WC larval comparisons were enriched in 45 and 46 categories, respectively. The categories that contained the highest number of DEGs in both comparisons were metabolic process, cellular process and protein binding. (Table S7 and S8). At the adult stage, DEGs from DC/QC and DC/WC drone comparisons were enriched in 40 and 47 categories, respectively (Table S9 and S10). Similarly, the categories that contained the highest number of DEGs were metabolic process, protein binding and membrane.

We compared the DEGs and in each GO category and each KEGG pathway, and the expressed honey bee genes in this study were used as background genes (Table S7, 8, 9, 10, 11, 12) to correct the enriched GO terms / pathways of DEGs. Interestingly, the categories with the highest rate (the rate was the number of DEGs divided by number of background genes in as same GO category) in the DC/QC and DC/WC larval comparisons were nutrient reservoir activity, structural molecule activity, electron carrier activity, growth, etc. (Table S7 and S8). At adult stage, the highest categories in the DC/WC drone comparison were nutrient reservoir activity, growth, reproduction, etc. (Table S9). The DC/QC drone comparison showed a slightly different result. The categories with the highest rate were cell killing, electron carrier activity, reproduction, locomotion, etc. (Table S10).

**Fig. 6:**
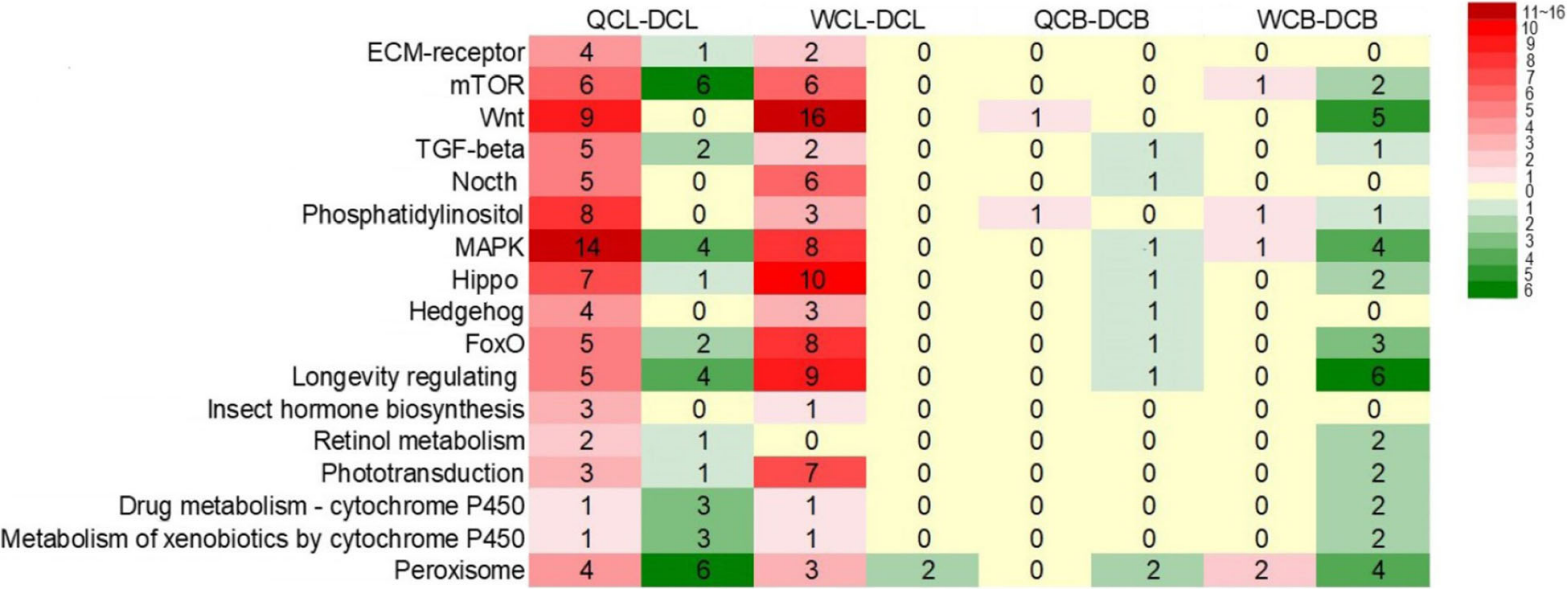
Number of DEGs between drones from male cells and female cells mapped to 17 important KEGG pathways. “QC-L”, “WC-L” and “DC-L” are queen-cell, worker-cell and drone-cell drone larvae respectively. “QC-A”, “WC-A” and “DC-A” are queen-cell, worker-cell and drone-cell adult drones respectively. Number of DEGs in each KEGG category were presented with color scales. Red color indicates number of up-regulated genes in female cell groups compared to male cell groups, whereas green color indicates number of down-regulated gene. Detailed information of these DEGs was presented in Table S11

KEGG enrichment analysis showed a high number of DEGs between drones from male cells and female cells at both larval and adult stages were enriched in Wnt and Notch signaling pathways, mitogen-activated protein kinase (MAPK), mammalian target of rapamycin (mTOR) pathway, Hippo signal pathway, insect hormone biosynthesis, longevity regulating pathway, cytochrome P450 metabolism, phototransduction-fly, etc. (Fig. 6; Table S11).

## Discussion

The environment has a profound effect on the development of many animals including eusocial insects, as a result of phenotypic plasticity [5, 6]. However, the effects of environmental factors on honeybee drone development and quality remain unclear. This study investigated the effects of honeybee female developmental factors on male development.

Our results showed that 3rd instar drones reared in female cells had thousands of DEGs, compared with natural drone larvae (Table 1), in which many were enriched in some important KEGG pathways, such as mTOR, Wnt, MAPK pathways and GO categories (metabolic process, nutrient reservoir activity, electron carrier activity and growth) (Fig. 6; Table S7, S8 and S11). The mTOR, Wnt, Notch, transforming growth factor-beta (TGF-β) and hippo signaling pathways play an essential role in developmental processes, such as caste differentiation, embryogenesis, morphogenesis, imaginal disc development and organ size regulation in honeybees and other insects [29–35]. These results demonstrated a clear difference in gene expression between honeybee male larvae developed from drone cells and female cells. In addition, the larval diets in QC and WC cells were significantly different with that in DC cells and the weight of 3rd instar drone larvae in female cells were also significantly lower than that in male cells (Fig. 1). At this stage, larvae from all three groups were small and their developmental space was large. Hence, the differences in gene expression between drone larvae developed from male cells and female cells, like biased gene expression in queen-worker differentiation [36], are possibly induced by differences in their diets.

Honeybees have a worker policing system so that worker-laid eggs can be identified and removed [37, 38], but this policing system is based on the pheromones on eggs marked by the mother queen [39]. In this study the drone eggs were all laid by queens, therefore, the worker policing system may not applicable. It is unclear whether workers could recognize drone larvae in queen and worker cells. In this study, the larval food remaining in QCs and WCs was significantly different compared to DC cells (Fig. 1). The remaining food amounts in QCs and WCs were consistent with that in natural queen and worker cells containing queen and worker larvae respectively [6, 7]. This suggests that nurses may not be able to recognize male larvae in female cells at early larval stage, and therefore deliver the female larval diets to drone larvae in female cells. The differences of gene expression (Table 1, DEGs: QC/DC:1761, WC/DC:889) among 3rd instar drone larvae from QC, WC and DC suggest that the food in female cells for young drone larvae should be distinct from that of natural drone larvae and can considerably affect the development of drones. A previous study showed that workers that emerged from DCs had larger acid glands and a higher number of ovarioles [20], which also revealed that the developmental conditions of honeybee drone larvae differ from worker larvae and influence the development of workers. In this study, we showed that honeybee drones developed from QCs and WCs also had considerable changes in their body size, reproductive tissues and gene expression compared to DC ones. These two studies [40, 41] revealed that honeybee female or male developmental environment could influence the development of their opposite sex.

More interestingly, QC drones showed significant differences in morphology, reproductive organs and gene expression, compared with the natural DC drones (Table 1; Figs. 2, 3 and 5). The QC drones were fed with royal jelly for 48 h during their early larval stage. Many DEGs were involved in olfaction, vision, growth, sex regulation, energy support, etc. (Fig. 5). Poor abilities in vision, olfaction and flying can cause a high failure rate in flying mating, as drones use visual and chemical cues to detect queens during mating flight [7]. Therefore, the honeybee female developmental environment could dramatically alter the gene expression of drones and influence their body development.

Royal jelly, which is fed to honeybee queen larvae, is different from worker jelly and drone jelly in minerals, vitamins, sugars, juvenile hormones and major royal jelly proteins [6, 8–11]. Queen larvae are fed more frequently with more food than workers and drones [6]. However, QC drones fed with richer food at early larval stage had a less developed body and reproductive organs than DC drones (Figs. 2 and 3), which suggests that honeybee royal jelly has a strong effect on male development. The royal jelly in QCs possibly delays drone development, although it is more nutritious than drone jelly.

The worker and drone jellies are physiologically equivalent, as exchanging their food results in normal drones [6, 19]. However, this study showed a notable difference in gene expression between 3rd instar C and WC drone larvae (Table 1; Fig. 4). Some DEGs between DCs and WCs are involved in mTOR, hormone biosynthesis, mitogen-activated protein kinase (MAPK), hippo, Wnt signaling pathways, etc. (Figures 5 and 6). These pathways participate in the regulation of insect development [30–36]. For example, the mTOR pathway is the central component of a conserved eukaryotic signaling pathway, regulating cell and organismal growth in response to nutrient status [42, 43]. Suppressing this pathway delays pre-adult development and reduces larval and adult body size in *Drosophila* [43]. It also plays a key role in the determination of honeybee queen-worker differentiation [44]. Therefore, the worker and drone jellies may not be fully equivalent, which influences the development of WC drones. This is supported by our results that drones developed from WCs resulted in smaller body size and less-developed reproductive organs (Figs. 2 and 3).

Moreover, we observed that workers increased the height of walls of WC drones but did not change the diameter of the cells, indicating that the cell size of WC drones is smaller than that of DC drones. Shi et al. showed that diet and cell size both contributed to the honeybee queen-worker caste differentiation [16]. According to Berg et al., cell size could strongly influence body size and weight of drones [21]. Consequently, the smaller cells size might be another factor contributing to the differences between WC and DC drones.

The present study and previous evidence [26] clearly revealed that honeybee males require different nutrition and developmental space compared to that of females during early development. This demonstrates that environmental conditions act as important factors in the developmental regulation of sex differentiation under haplo-diploid system. Since most of the studies focused on environmental factors manipulating animal development in terms of body mass, metabolism, reproduction, diseases, behavior and longevity [45, 46], the factors modulating sex differentiation were rarely discussed. Therefore, this study enriches our understanding on the interaction between environmental factors and sex differentiation in social insects. Further studies could use special tissues such as reproductive tissues for RNA-Seq and perhaps more specific differences would be detected among QC, WC and DC drones.

Moreover, drones contribute half of the DNA to breeding populations and play a vital role in species reproduction [7]. High quality drones increase the fitness of the whole honeybee colony and the next generations [7]. Previous studies have reported that larger and heavier drones have larger mucus glands and a higher volume of semen, and these produce more spermatozoa, show fewer sperm abnormalities [27, 47, 48, 49]. There is also a positive correlation between body size and the reproductive success of honeybee drones [21, 24]. This study indicates that honeybee female developmental environment reduced drone body size and developmental level of reproductive organs, and induced a large number of DEGs (Figs. 2, 3, 4, 5 and 6; Table 1), resulting in low-quality drones. In fact, ovigerous workers from a queen-less colony or non-mated queens lay unfertilized eggs in WCs, and those eggs could successfully be turned into drones, which are developed from female cells. A previous study [51] showed that WC and DC drones were equally likely to be accepted by workers in a honeybee colony, and a preference was shown it tended to favor WC drones. This may be a competition for high-quality drones during mating flights. If these dysplasia drones continuously mate with virgin queens, the fitness of honeybee colonies will be decreased. Therefore, this study offers a caution to the honeybee breeding industry to prevent interference from dysplasia drones.

## Conclusions

In conclusion, this study demonstrated that honeybee female developmental environment, such as nutrition and cell size, have a strong effect on male development. This study serves as a model for understanding the modulation of environmental factors on sex differentiation and developmental plasticity in social insects, which is overlooked. Further investigations are needed to explore the adverse effects of dysplasia drones on the fitness of the whole honeybee colony and next generations.

## Methods

### Animals and experimental design

Three honeybee (*Apis mellifera*) colonies were used in this study, and each colony had a mated queen and about 30,000 bees. They were kept in the Honeybee Research Institute, Jiangxi Agricultural University, China.

Queens were caged onto a drone frame to lay eggs for 6 h. After hatching for 6 h, drone larvae were moved to worker cells and commercial plastic queen cells, and the rest of larvae remained in drone cells. We transplanted these drone larvae to drone cells after feeding with royal jelly in QCs for 48 h. The DC and QC drones finally were emerged from drone cells, and the WC drones were emerged from WCs.

We collected 3rd instar drone (around 54 h old larvae) of QCs, WCs and DCs, respectively, for RNA-SEq. Each sample contained 6 larvae, and each group had 3 biological replicates from three colonies. Newly emerged drones from these three groups were collected for morphological measurement and RNA-SEq. For RNA-Seq, each sample contained 2 newly emerged drones, and each group had three biological replicates. All samples for RNA-Seq were put into liquid nitrogen, frozen for 30 min and then stored at -80℃ before RNA extraction. For morphological measurement, each group had 15 newly emerged drones from three colonies. For weight measurement, each group had 90 replicates.

To measure the food remaining in female cells at the young larval stage, queens were caged on an empty drone frame to lay eggs for 6 h. Eggs were grafted into queen and worker cells before hatching respectively. The eggs that remained in drone cells were used as controls. The weight of food remaining in three types of cells at 24 h, 48 h, and 72 h was measured using an analytical balance (accuracy: 0.01 mg, HZ-104/35S, USA.HZ Co. Ltd.).

### Morphological measurement

Morphological data included birth weight, length and width of drone wings, width of thoraxes and horizontal area of head. Drone frames were placed into an incubator for 12 h before emerging. Newly emerged drones were captured and weighed using an analytical balance (0.01 mg, AUY120, Shimazu Co. Ltd., Japan). Subsequently, drones were anesthetized by CO_2,_ and their wing length and width, thorax width and head horizontal area were measured by a microscopic imaging system (microscope: GL99TI, Guiguang Co. Ltd., China; Troup view software: x64, ToupTek Co. Ltd., China). The data of head horizontal area were obtained using Image J (1.52a, Wayne Rasband National Institutes of Health, USA) using images taken by the above microscopic imaging system (microscope: GL99TI, Guiguang Co. Ltd., China; Troup view software: x64, ToupTek Co. Ltd., China).

The drone reproductive organs, including seminiferous tubules length, horizontal area of seminal vesicles and mucous glands, were measured. Firstly, the drones were dissected, and the testes, seminal vesicles and mucous glands were taken out and washed with saline solution and placed on glass slides. After removing redundant tissues, the location of the tissues was adjusted on glass slides, and the images were taken under the microscopic imaging system. The tunica testis of the testes was removed, and seminiferous tubules were divided into many small aggregations according to the method developed by Tavares et al. [25]. The horizontal area of seminal vesical and mucous glands was measured by Image J, and the length of seminiferous tubules was measured by Troup view.

### RNA extraction and sequencing

Total RNA of newly emerged drones and 3rd instar of those three groups was extracted in accordance with the standard protocol of the TRlzol Reagent (Life Technologies, CA, USA), respectively. RNA integrity and concentration were tested by an Agilent 2100 Bioanalyzer (Agilent Technologies, Inc., CA, USA).

RNA-Seq was done according to our previous study [51]. Briefly, mRNA of each sample was isolated from total RNA by a NEBNext Poly (A) mRNA Magnetic Isolation Module (NEB, E7490; New England Biolabs Inc., USA) and broken randomly using Fragmentation Buffer. Afterward, a cDNA paired-end library of each sample was constructed using a NEBNext Ultra RNA Library Prep Kit (NEB, E7530; New England Biolabs Inc.,USA) and the NEBNext Multiplex Oligos (NEB, E7500 ; New England Biolabs Inc.,USA). Purified double-stranded cDNA was isolated by AgencourtAMPure XP beads (Beckman Coulter, Inc.) and acquired by PCR. The effective concentration of the library (> 2nM) was accurately quantified by qRT-PCR to ensure the quality of the library. All constructed cDNA libraries were sequenced by the HiSeq X Ten sequencing platform (Illumina Inc., USA).

### Analysis of Pearson`s correlation

The reliability of biological replicates in each group was evaluated using Pearson’s correlation coefficient analysis. The rate of Pearson’s correlation coefficient over 0.8 was considered as a conventionally accepted threshold for valid replicates [52]. One 3rd instar drone sample with a very low rate of Pearson’s correlation coefficient (< 0.8) was removed before gene expression analysis (Fig. S1).

### Gene expression analysis

Like our previous study [52], low-quality reads were filtered and those with a sequencing error rate of less than 1 % (Q20 > 98 %) were retained. The clean reads were mapped to the newest version of honeybee genomics (Amel_HAv3.1). Gene expression levels were estimated using fragments per kilobase of exon per million fragments mapped (FPKM) values by the Cufflinks software [52].

DESeq2 [53] was used to analyze the differential expression among three drone groups using gene read counts rather than FPKM values. Fold Change ≥ 1 and *p* < 0.05 were used as the screening criteria to identify significant DEGs among three groups. We selected 61 and 50 of interest genes that are involved in caste differentiation and development regulation [30–36] from comparisons at larval and adult stages respectively. The log10 fold change values of these genes were used for heatmap analysis in R package (4.0.2), and the results were shown in Figs. 4 and 5.

### Enrichment of GO and KEGG

Sequences of DEGs from all comparisons were against various protein and nucleotide sequence databases by BLASTX (version 2.2.28), including the National Center for Biotechnology Information (NCBI) non-redundant protein (Nr) database and Swiss-Prot database and non-redundant nucleotide sequence (Nt) database with a cut-off E-value of 10^− 5^. DEGs were mapped in terms of the GO database and a hypergeometric test [54] (*p* < 0.05 indicates the significance) in GO enrichment analysis to identify their significantly enriched GO terms.

Similarly, all DEGs from each comparison were mapped to the KEGG protein database (http://www.genome.jp/kegg/kegg1.html) using BLAST (E-value < 1e-5). The statistical enrichment of DEGs in KEGG pathways was analyzed by a hypergeometric test (Q-value < 0.05) using the KOBAS 2.0 software [55].

### qRT-PCR analysis of ten selected genes

Total RNA of 3 d larvae and newly emerged drones from DCs, QCs and WCs were extracted and used for qRT-PCR validation of RNA-Seq data. The purity (260nm/280nm ratio between 1.8 and 2.0 for RNA) and the concentration of each RNA sample were measured according to our previous study [56]. RNA samples were standardized for reverse transcription. cDNA was synthesized using MLV reverse transcriptase (Takara, Japan), according to the manufacturer’s instructions. The β-action gene was used as an internal housekeeping gene. A total of 10 genes from RNA-Seq results were randomly selected as target genes for qRT-PCR analysis (Fig. S2 and S3). Primers for these genes were designed using Primer 5.0 software (Table S12). qRT-PCR cycling conditions were as follows: 94 °C for 2 min, 40 cycles, followed by 94 °C for 15 s, 60 °C for 30 s, and 72 °C for 30 s. The specificity of the PCR products was verified by melt curve analysis for each sample. For each gene, three biological replicates (with five technical replicates for each biological replicate) were performed. Control and target genes for each sample were run on the same plate to control for inter-plate variation. The Ct value for each biological replicate was obtained by calculating the mean of three technical replicates. The relative expression level among DC, QC and WC larvae or newly emerged drones was calculated using the 2^−ΔΔCt^ formula reported by Liu and Saint [57].

### Data analysis

Data were firstly verified its assumption about normality by the Shapiro-Wilk test before ANOVA or T test, and all tested data were accorded to normality description (*p* > 0.05). Data of larval food from three treatments (Fig. 1) were compared using one-way ANOVA test and adjusted with Bonferroni correction, and *p* value < 0.025 considered as significant difference. Data of morphological indexes and reproductive tissues among three groups (Figs. 2 and 3) were analyzed by Independent-Sample T test. The critical *p* values of were adjusted to 0.0167 according to the Bonferroni correction (SPSS 22.0.0.0. IBM Corporation, USA). The *p* value < 0.0167 was considered as significant difference. The relative expression levels of 10 genes in qRT-PCR experiment were calculated using 2^−ΔΔCt^ format, and then compared with RNA-Seq results.

## Supplementary Information

**Additional file 1:** Supplementary figures. This file has included a set of 3 supplementary figures providing: Pearson’s correlation coefficient analysis of 18 samples (**Figure S1**); Expression of 10 genes in three comparisons at 3rd instar stage by qRT-PCR (Figure S2); Expression of 10 genes in three comparisons at newly emerged stage by qRT-PCR (**Figure S3**).

**Additional file 2:** Supplementary tables. This file has included a set of 12 supplementary figures providing: Statistics of sequencing data (**Table S1**); Statistical table of sample sequencing data and sequence alignment of selected reference genomes (**Table S2**); DEGs between QC and DC larvae (**Table S3**); DEGs between WC and DC larvae (**Table S4**); DEGs between QC and DC drones (**Table S5**); DEGs between WC and DC drones (**Table S6**); DEGs between QC and DC larvae enriched in GO enrichment (**Table S7**) DEGs between WC and DC larvae enriched in GO enrichment (**Table S8**); DEGs between QC and DC drones enriched in GO enrichment (**Table S9**); DEGs between WC and DC drones enriched in GO enrichment (**Table S10**); DEGs between drones from male cells and female cells enriched in 17 important KEGG pathways (**Table S11**, the data is the raw data supporting the Fig. 6); qRT-PCR primer sequences (**Table S12**).

## Data Availability

We confirm that all relevant data are included in the article and its supplementary information files. The raw data of honeybee larval food and morphological data are uploaded on Dryad database: 10.5061/dryad.v9s4mw6v1. The transcriptome data of 18 samples are uploaded on SRA with accession numbers as follow. RNA-Seq raw data of 3^rd^ instar drone: WCs: NCBI SRA: SRR12031955; SRR12031954; SRR12031956. QCs: NCBI SRA: SRR12031970; SRR12031969; SRR12031960. DCs: NCBI SRA: SRR12031959; SRR12031958; SRR12031957. RNA-Seq raw data of adult drones: WCs: NCBI SRA: SRR12031953; SRR12031968; SRR12031967. QCs: NCBI SRA: SRR12031966; SRR12031965; SRR12031964. DCs: NCBI SRA: SRR12031963; SRR12031962; SRR12031961.

## Abbreviations

QC: Queen-cell drone
WC: Worker-cell drone
DC: Drone-cell drone
DEG: Significantly differentially expressed genes
RNA-Seq: RNA sequencing
GO: Gene ontology
KEGG: Kyoto encyclopedia of genes and genomes
MAPK: Mitogen-activated protein kinase
mTOR: Mammalian target of rapamycin
TGF-β: transforming growth factor-beta
FPKM: Fragments per kilobase of exon per million fragments mapped
