# Supplementary figures and images for: Female developmental environment delays development of male honeybee (Apis mellifera)
Source: BMC Genomics. 2021 Sep 27;22:699. doi: 10.1186/s12864-021-08014-1 (PMC8477528; doi:10.1186/s12864-021-08014-1)

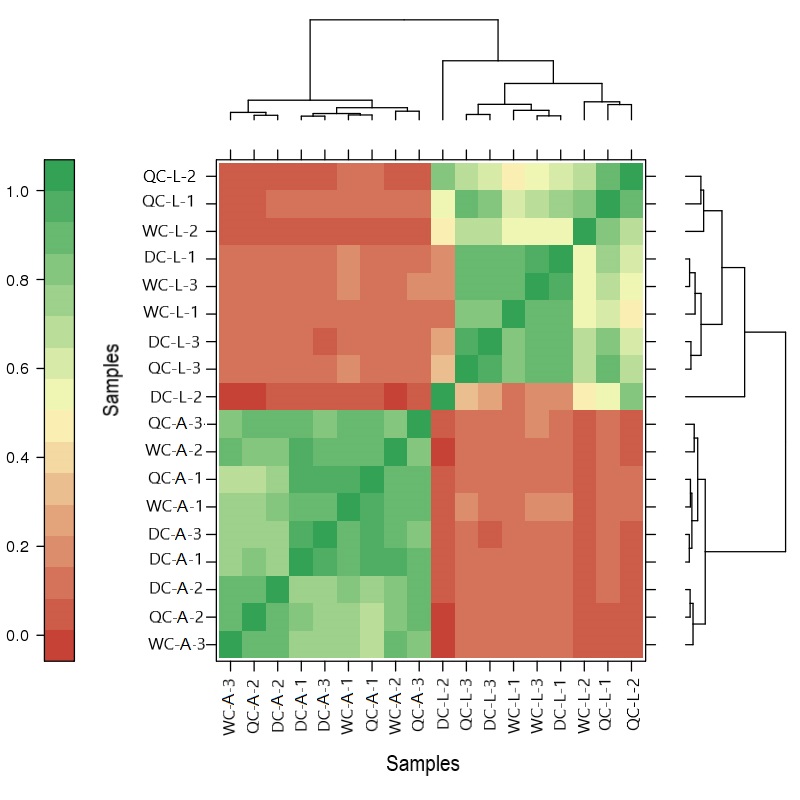

Supplement: Supplementary file 1 — Additional file 1: Supplementary figures. This file has included a set of 3 supplementary figures providing: Pearson’s correlation coefficient analysis of 18 samples (Figure S1); Expression of 10 genes in three comparisons at 3rd instar stage by qRT-PCR (Figure S2); Expression of 10 genes in three comparisons at newly emerged stage by qRT-PCR (Figure S3). [file 12864_2021_8014_MOESM1_ESM.zip › Fig S1.jpg]

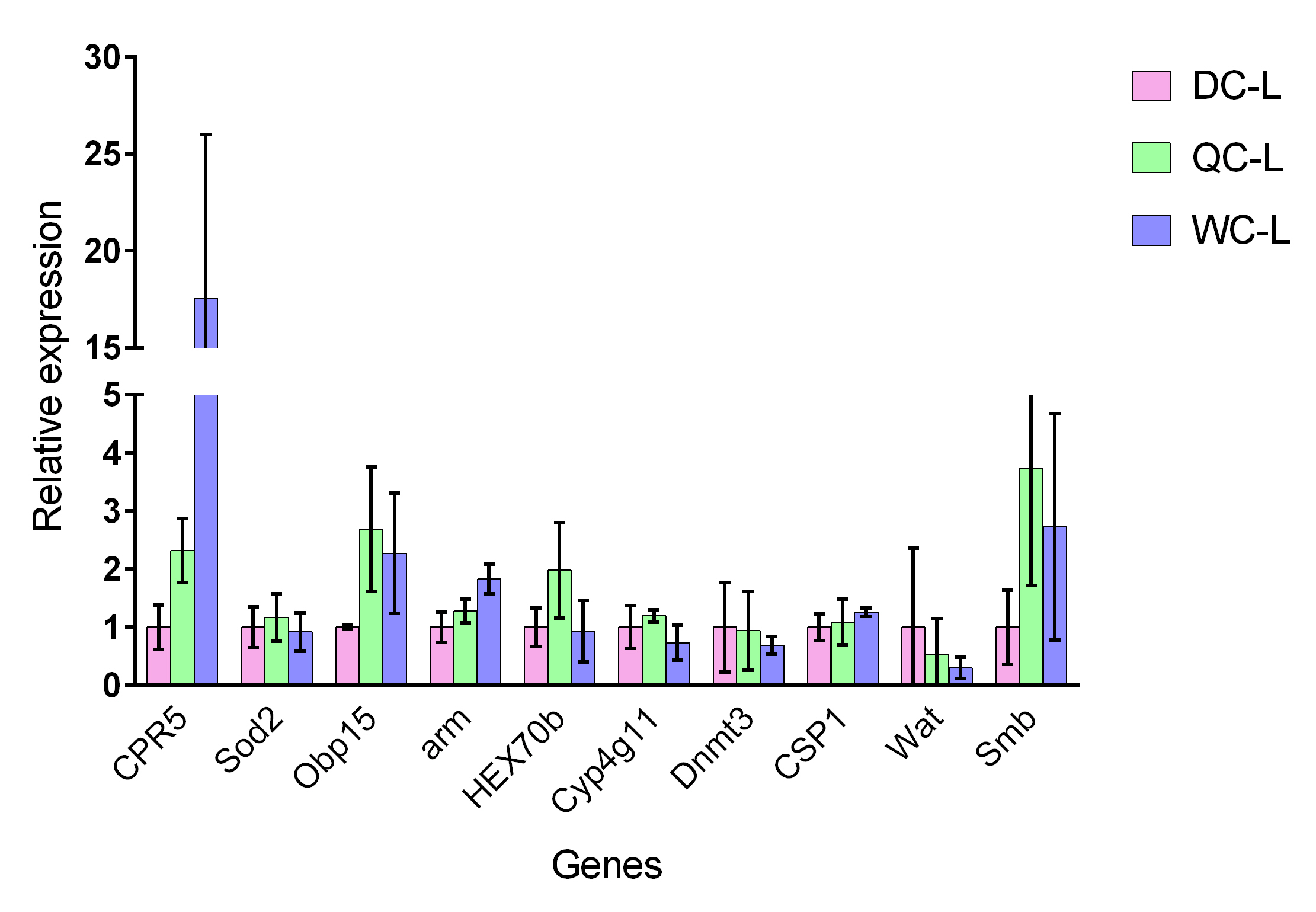

Supplement: Supplementary file 1 — Additional file 1: Supplementary figures. This file has included a set of 3 supplementary figures providing: Pearson’s correlation coefficient analysis of 18 samples (Figure S1); Expression of 10 genes in three comparisons at 3rd instar stage by qRT-PCR (Figure S2); Expression of 10 genes in three comparisons at newly emerged stage by qRT-PCR (Figure S3). [file 12864_2021_8014_MOESM1_ESM.zip › Fig S2.jpg]

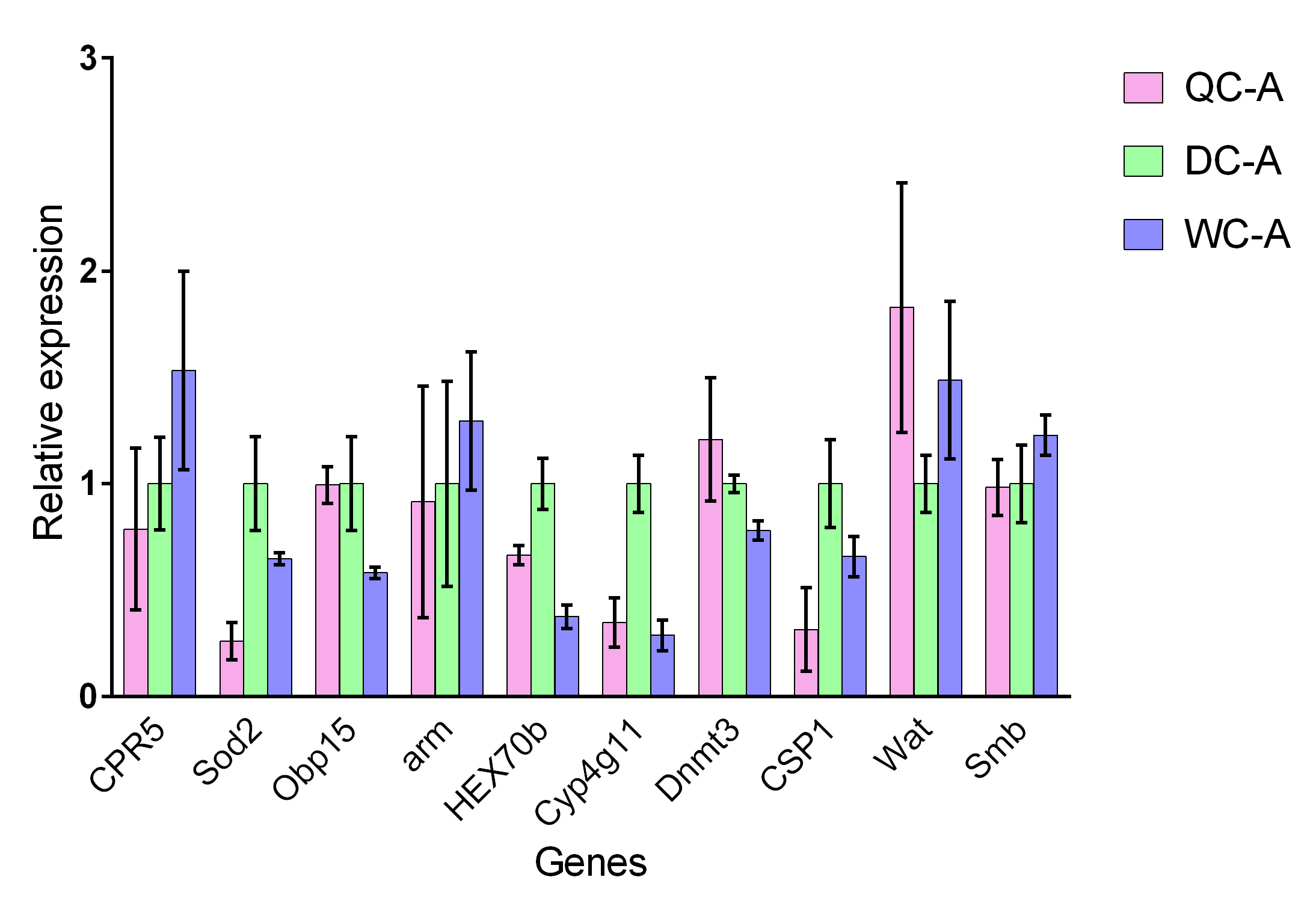

Supplement: Supplementary file 1 — Additional file 1: Supplementary figures. This file has included a set of 3 supplementary figures providing: Pearson’s correlation coefficient analysis of 18 samples (Figure S1); Expression of 10 genes in three comparisons at 3rd instar stage by qRT-PCR (Figure S2); Expression of 10 genes in three comparisons at newly emerged stage by qRT-PCR (Figure S3). [file 12864_2021_8014_MOESM1_ESM.zip › Fig S3.jpg]
